# Conquering Mount Fuji: Resolution of Tension Pneumocephalus with a Foley Urinary Catheter

**DOI:** 10.1155/2011/164316

**Published:** 2011-07-02

**Authors:** Shahid M. Nimjee, Ali R. Zomorodi, D. Cory Adamson

**Affiliations:** Division of Neurosurgery, Duke University Medical Center, Durham, NC 27710, USA

## Abstract

Tension pneumocephalus is the presence of air or gas in the cranium that is under pressure. It occurs due to disruption of the skull, including trauma to the head or face, after neurosurgical procedures and occasionally, spontaneously (Schirmer et al., 2010). Patients typically present with headache but can also have neurological deficits such as decreased mental status, numbness, and weakness (Schirmer et al., 2010). It is diagnosed by computerized tomography (CT) scan (Michel, 2010). The characteristic finding is that the two frontal poles of the brain are separated by air. After diagnosis, treatment is imperative for both symptomatic relief and preventing further compression. We present a case of a patient who presented with tension pneumocephalus and unconventional treatment that resulted in clinical improvement of his symptoms and radiographic resolution of his condition.

A 62-year-old male underwent a transcranial approach to remove an intraorbital hemangioma. He did well in the immediate postoperative period. He returned to the emergency department 8 days later complaining of a severe headache and swelling around his head. On physical exam, his wound was intact; however, the area was raised. He had no neurological deficits [1, 2]. 

A CT scan (Figure 1(a)) revealed a collection of air in both the subgaleal and subdural spaces. The imaging revealed the classic Mount Fuji sign, favoring the right side, where the craniotomy had taken place.

There were no external ventricular drain catheters available in the hospital. We prepared the area of interest with chlorhexidine and in sterile fashion, made a stab-incision, slightly posterior to his previous incision and inserted a Foley catheter in the subgaleal space. His subgaleal collection resolved, and his right forehead was flat again. Twenty minutes later, he stated that his headache had resolved. 

A CT scan the following day revealed the presence of the Foley catheter and marked improvement in his pneumocephalus (Figure 1(b)). We removed the catheter 2 days later. One month later, his pneumocephalus had completely resolved (Figure 1(c)).

Compared to an external ventricular drain (EVD) catheter, which has a diameter 1.5 mm, a 14 French Foley catheter is 4.622 mm. It therefore required a larger incision than an EVD catheter. There was, however, no increased risk in placing the catheter in the subgaleal space compared to an EVD catheter, and in this case it relieved the patient of his symptoms and resulted in complete resolution of his tension pneumocephalus.

## Figures and Tables

**Figure 1:**
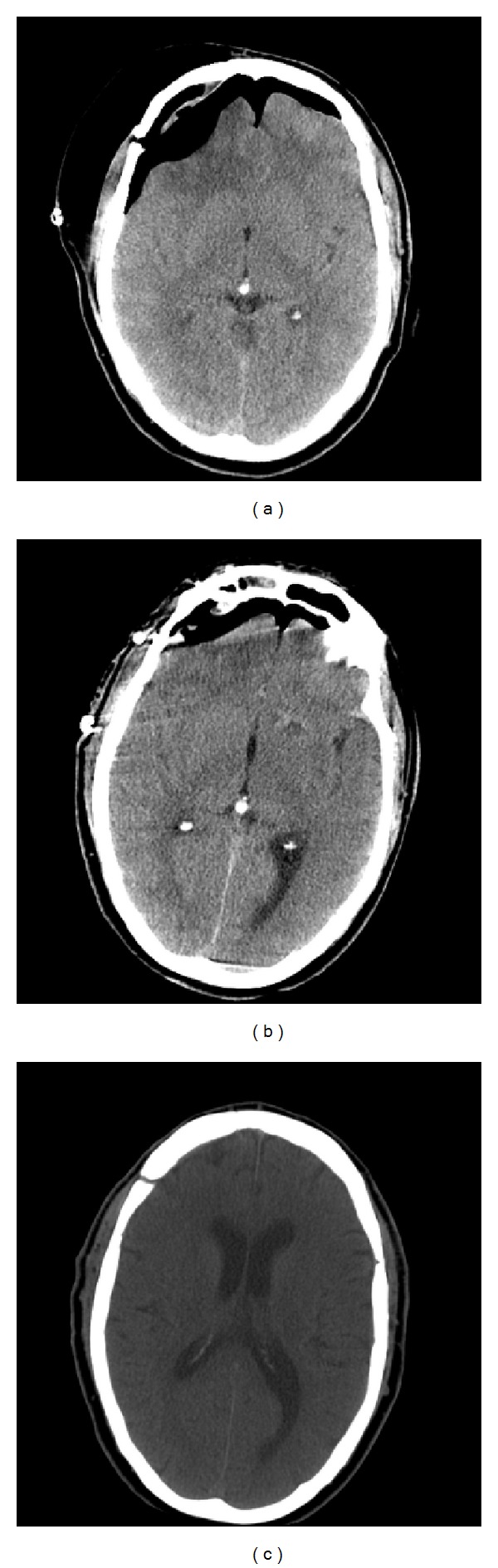
Computerized tomography scans (a) on presentation to the emergency department. There is a subgaleal collection of air in addition to the tension pneumocephalus. (b) After placing the Foley catheter, the subgaleal collection has completely resolved and the tension pneumocephalus has significantly improved. The patient's symptoms had resolved after Foley placement. (c) 1 month later showing complete resolution of tension pneumocephalus.
